# Treatment for clear cell carcinoma of the abdominal wall at a tertiary cancer center

**DOI:** 10.1038/s41598-022-14917-0

**Published:** 2022-06-25

**Authors:** Zheng Feng, Hao Wen, Xingzhu Ju, Rui Bi, Xiaojun Chen, Wentao Yang, Xiaohua Wu

**Affiliations:** 1 Department of Gynecological Oncology, Fudan University Shanghai Cancer Center, 270 Dong-an Road, Shanghai, 200032 China; 2 Department of Oncology, Shanghai Medical College, Fudan University, Shanghai, 200032 China; 3 Department of Pathology, Fudan University Shanghai Cancer Center, Shanghai, 200032 China

**Keywords:** Cancer, Oncology

## Abstract

Clear cell carcinoma (CCC) of the abdominal wall is a rare and agressive disease. We aim to elucidate the clinical and prognostic characteristics of this disease. Medical records of ten patients diagnosed with CCC of the abdominal wall at Fudan University Shanghai Cancer Center were reviewed. We illustrate the clinical characteristics, treatment modality, and development of local recurrence or distant metastasis, as well as the survival outcome. The median (range) age of patients was 47 (39–61) years old. All patients had a history of cesarean section and abdominal wall endometriosis. All patients had primary surgery before referred to our center. Seven patients had only tumor resection, while two patients had lymph node metastasis at primary diagnosis. Four patients underwent supplementary surgery, and all postoperative pathology were negative. Genetic analyses had also been performed. The median (range) follow-up time was 20 (12–59) months. Local recurrence and lymph node metastasis were the most common recurrence types. The median (95% confidence interval) PFS was 11 (8.08–13.92) months. In summary, primary surgery should consider wide tumor resection and lymph node dissection. Adjuvant chemotherapy and radiotherapy should be recommended for potential benefits. More cases are still needed to elucidate the clinical management of this disease.

## Introduction

Clear cell cancer (CCC) of the abdominal wall is an extremely rare disease. To date, only a small number of cases have been reported since the first documentation in 1986^1^. It is plausible that CCC of the abdominal wall originates from malignant transformation of abdominal wall endometriosis^2^. And implantations of ectopic endometrium during a prior history of gynecological surgery, especially cesarean section, was the main origin^3^.

Due to its rarity, the management of abdominal wall CCC is not well established. Patients were treated with different strategies, including surgery, chemotherapy and radiotherapy. However, individualized case report could not illustrate the characteristics of this disease. Therefore, we comprehensively reviewed the characteristics of ten cases with this disease at our institution in the past 5 years. To our knowledge, it is the largest cohort to date. Here, we summarized the treatment and recurrence patterns, as well as genetic data, to provide more information of this rare condition.

## Methods

### Clinical data

The data are anonymous, and the requirement for informed consent was therefore waived (Committee at Fudan University Shanghai Cancer Center, IRB number: 050432-4-1212B).

Medical records of all patients treated for CCC of the abdominal wall at our institution between January 2015 and December 2020 were reviewed. Ten cases were confirmed via pathological review by two experienced gynecologic pathologists.

Clinical data including age, treatment strategy and patients’ disease status were obtained from medical records and cancer registries. Progression-free survival (PFS) was defined as the time interval from the date of diagnosis to the date of disease progression or recurrence, or the date of last follow-up with no relapsed disease. Overall survival (OS) referred to the time interval from the date of primary diagnosis to the date of death or the last follow-up (January, 2021).

Four patients had genetic tests. Paired peripheral blood (or normal tissue) and tumor samples were taken at the hospital and sent to the laboratory, where DNA extraction, targeted DNA sequencing, variant calling, and interpretation were performed.

### Statistical analyses

We used SPSS software (version 21.0) and GraphPad Prism (version 6.0) for the statistical analyses. Demographic data were described as the medians with ranges or the frequencies with percentages. The PFS were illustrated with the Kaplan–Meier curve.

### Ethical statement

This study was conducted according to the Declaration of Helsinki, and it was approved by the Committee at Fudan University Shanghai Cancer Center. The authors are accountable for all aspects of the work in ensuring that questions related to the accuracy or integrity of any part of the work are appropriately investigated and resolved.

## Results

The clinical and pathological outcomes of each patients are listed in Table 1. The median (range) age of the ten patients was 47 (39–61) years old. All patients had a history of cesarean section and abdominal endometriosis. Since our institution is a tertiary cancer center, all patients had primary surgeries before referred to our center. Seven patients had only tumor resection with so-called “clear margin”. One had pelvic lymph node metastasis, and another had inguinal lymph node metastasis at primary surgery. The CA125 levels were normal in nine patients, except case 7.

**Table 1:** Clinical and pathological outcomes of patients with clear cell carcinoma of the abdominal wall.

Cases	Age	Primary diagnosis	Primary pathology	Treatment strategy	Surgical patterns	Supplementary pathology
1	48	Tumor resection	Abd wall CCC	Supplementary surgery + CT	TAH + BSO + omentectomy	Negative
2	44	Tumor resection	Abd wall CCC	CT		
3	49	Tumor resection	Abd wall CCC	Supplementary surgery + CT	TAH + BSO + omentectomy	Negative
4	46	Tumor resection	Abd wall CCC	Supplementary surgery + CT	TAH + BSO + omentectomy	Negative
5	49	Tumor resection	Abd wall CCC	CT		
6	61	Tumor resection, TAH + BSO + omentectomy	Abd wall CCC, Gyn-	CT		
7	59	Tumor resection, Inguinal LND	Abd wall CCC, LN +	CT		
8	42	Tumor resection, TAH + BSO, BPLND	Abd wall CCC, Gyn-, LN +	CT		
9	42	Tumor resection	Abd wall CCC	RT		
10	39	Tumor resection	Abd wall CCC	Supplementary surgery + CT	TAH + BSO + omentectomy + BPLND	Negative

*CT* chemotherapy, *RT* radiotherapy, *TAH* total abdominal hysterectomy, *BSO* bilateral salpingo-oophorectomy, *Abd* abdominal, *Gyn* gynecological organs, *LN* lymph node, *BPLND* bilateral pelvic lymph node dissection.

We advised the seven patients with only tumor resection for supplementary surgery. Four patients underwent surgeries with negative postoperative pathologies (Table 1). Three patients refused additional surgeries and received either direct chemotherapy or radiotherapy. Among them, one had metastatic disease during adjuvant chemotherapy, and another one receiving adjuvant radiotherapy suffered from local recurrence at the third months (Table 2).

**Table 2:** Treatment outcomes of patients with clear cell carcinoma of the abdominal wall.

Cases	Treatment response	Recurrence pattern	PFS (month)	OS (month)	Status at last follow-up
1	CR	NED	46	46	NED
2	PD	Local recurrence, LN and pelvic metastasis	–	12	Dead
3	CR	Local recurrence	14	14	Secondary surgery
4	CR	Local recurrence	11	18	NED
5	CR	Local recurrence	22	24	Secondary surgery
6	CR	Local recurrence and LN metastasis	10	59	Dead
7	CR	LN metastasis	9	47	Salvage chemotherapy
8	CR	LN metastasis	6	13	Salvage chemotherapy
9	CR	Local recurrence	3	34	Salvage chemotherapy
10	CR	Local recurrence	15	16	Salvage chemotherapy

*CR* complete response, *PD* progressive disease, *PFS* progression-free survival, *OS* overall survival, *NED* no evidence of disease.

**Figure 1:**
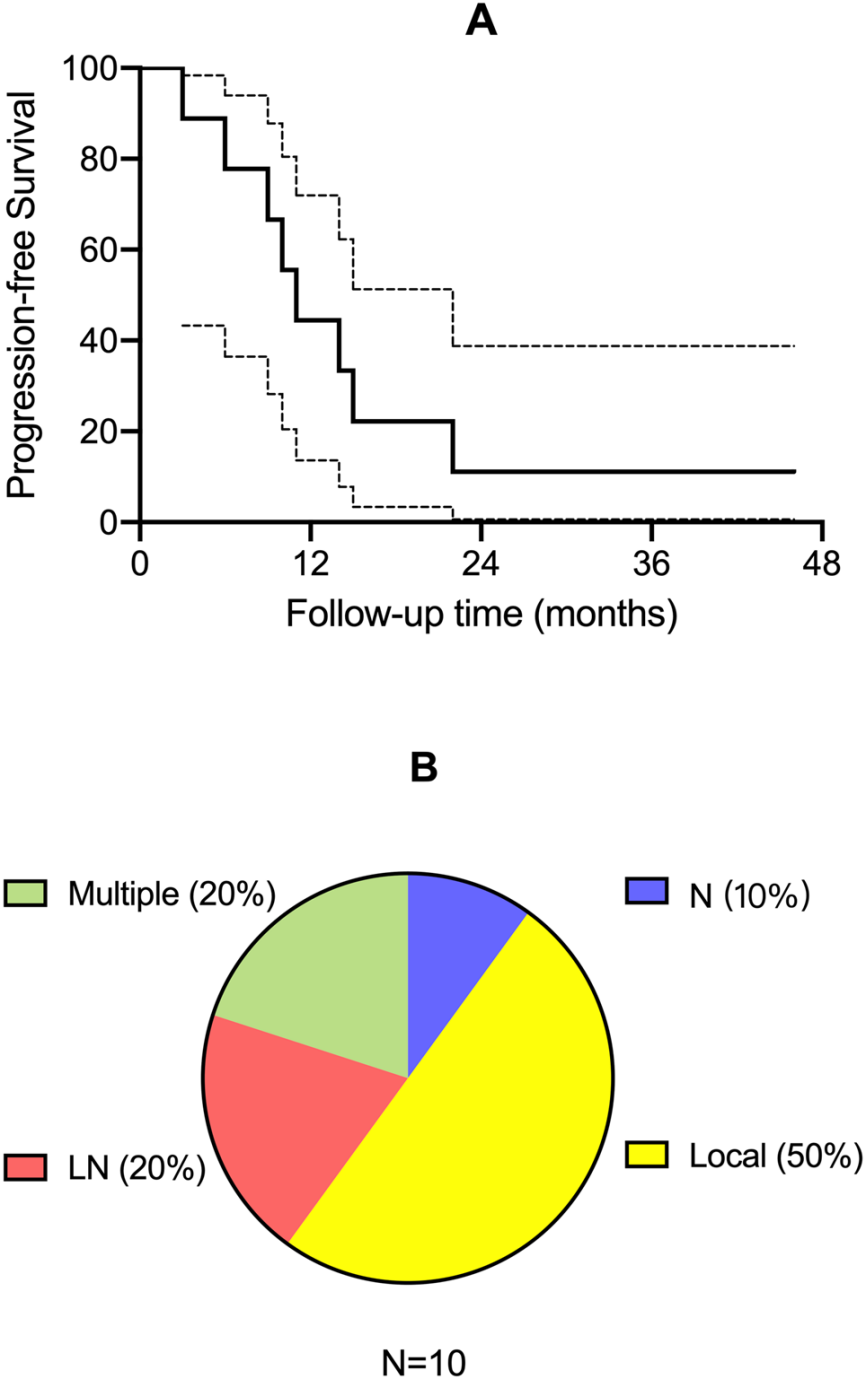
Progression-free survival and recurrence patterns of abdominal wall clear cell cancer. **(A)** Kaplan–Meier curve of progression-free survival for abdominal wall clear cell cancer. (**B**) Distribution of relapse sites, including local recurrence, LN (lymph node metastasis) and multiple sites. N represents no recurrence.

The median (range) follow-up time was 20 (12–59) months. Nine patients received adjuvant chemotherapy with paclitaxel and carboplatin. The median (95% confidence interval) PFS was 11 (8.08–13.92) months, and the recurrence patterns were also investigated (Fig. 1). Local recurrence was the most common type, and patients also tended to have lymph node metastasis despite negative at primary diagnosis (Table 2). Two patients died of this disease.

**Figure 2:**
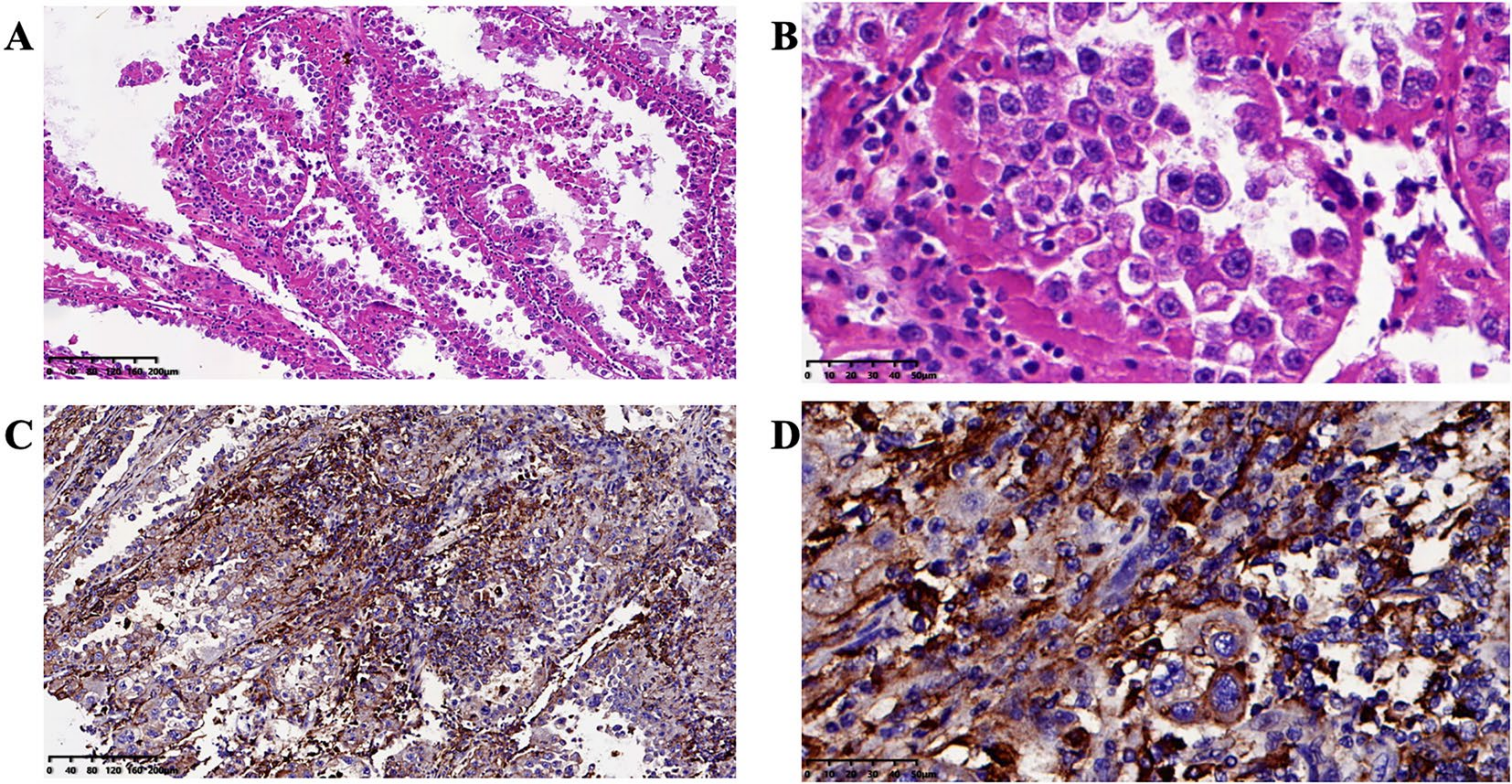
Photomicrography: microscopic results of abdominal wall clear cell cancer. **(A)** Hematoxylin and eosin stain with magnification ×100. (**B**) Hematoxylin and eosin stain with magnification ×400. (**C**) PD-L1 immuno-histochemical stain with magnification ×100. (**D**) PD-L1 immuno-histochemical stain with magnification ×400.

Four patients had genetic tests as reference (Table 3). Unfortunately, there were no valuable information for existing targeted therapy. For immunotherapy, mismatch repair deficiency, microsatellite instability and tumor mutation burden were also tested. However, the results were unsatisfactory. PD-L1 immuno-histochemical test had also been performed (Fig. 2). Case 2 had PD-L1 tumor positive score of 40%, and combined positive score of 40. However, she did not respond to PD-1 salvage therapy, and died 12 months after diagnosis.

**Table 3:** Genetic analyses of 4 patients with clear cell carcinoma of the abdominal wall.

Cases	Tier II gene variants	dMMR	MSI	TMB
2	ARID1A	–	MSS	3.33
3	TP53 and ARID1A	–	MSS	1.79
4	–	–	MSS	2.51
5	–	–	MSS	0.67

Tier II gene variants: variants of potential clinical significance.

*dMMR* mismatch repair deficiency, *MSI* microsatellite instability, *MSS* microsatellite stability, *TMB* tumor mutation burden.

## Discussion

Clear cell carcinoma (CCC) of the abdominal wall is exceptionally uncommon in clinical practice, with only about 40 cases having been reported in the literature^4,5^ (Table S1). Data based on individual cases could not illustrate the characteristics of this disease. Our study comprehensively reviewed a series of ten cases at our institution in the past 5 years. To our knowledge, it is the largest study to date, to provide more information of this rare disease.

Different from previous reports, patients had primary surgeries before referred to our institution. We tended to manage it according to ovarian CCC considering its origin from ectopic endometriosis. The surgical procedure includes wide tumor resection, hysterectomy, bilateral salpingo-oophorectomy, as well as omentectomy. Similar with previous cases, there were no evidence of malignancy in omentum^6,7^. Thus, omentectomy would be omitted except its metastasis or adhesion to the tumor mass. Hysterectomy and bilateral salpingo-oophorectomy could be performed for persistent pelvic endometriosis among menopausal women. However, no evidence of disease was found in our resected pelvic organs. Also, the rate of pelvic metastasis was low according to literature, and only one bladder invasion reported^8^. Thus, the necessity for resecting gynecologic organs need further investigation, especially for the prognostic implication.

Inguinal and pelvic lymph nodes were the most common metastatic sites. Lymphadenectomy is mainly performed as part of a debulking procedure in literature^9^. The only one with lymphadenectomy in our series showed no evidence of metastasis. However, three out of six patients with clinical and radiological negative lymph nodes were confirmed pelvic lymph node metastasis on final pathologal diagnoses^5,6,10–13^. Gentile et al.^10^ reported a case with both inguinal and pelvic lymphadenectomy and had only pelvic lymph node metastasis. Thus, lymphadenectomy should be performed at primary surgery.

Some might suggest chemotherapy before the primary surgery to reduce tumor size and surgical difficulty. However, according to literature, patients with abdominal CCC did not response to neoadjuvant chemotherapy^5,6,14,15^. Thus, surgery had its priority in the treatment of abdominal CCC.

The role of radiotherapy in abdominal CCC has been under debate. Miller et al.^16^ reported that one abdominal CCC patient received surgery combined with adjuvant chemotherapy and radiotherapy, and achieved no relapse after 60 months. Ruiz et al.^7^ reported two cases, the one without radiotherapy suffered a local recurrence 6 months after adjuvant chemotherapy alone. Harry et al.^17^ and Mert et al.^6^ also reported successfully treatment by adjuvant radiotherapy. While in our cohort, the patients receiving radiotherapy alone relapsed locally within three months. This may be due to the unknown tumor margin. Adjuvant chemo-radiotherapy was associated with a lower risk of disease failure compared with chemotherapy alone in early stage ovarian CCC treatment^18^. Regarding the similar origin of ovarian CCC, radiotherapy could help for regional disease control.

Besides, chemotherapy could not be avoided for abdominal CCC treatment. Local recurrence and lymph node metastasis were the main recurrence patterns in our cohort. Although hematogenous metastasis was not observed in our cases, it is not rare in literature. Liver, lung and bone metastasis had all been reported^5,15,19,20^. Postoperative adjuvant chemotherapy regimen was consistent in literature, with paclitaxel and carboplatin.

In addition, we performed genetic analyses of abdominal CCC for the first time. Although the results were unsatisfactory, it could provide more information on the pathogenesis and biological behavior for this rare disease.

Our study has included the largest number of abdominal CCC patients, though only ten patients in the cohort. Nevertheless, we would like to share our clinical experience to help to determine its clinical course and prognosis.

## Conclusions

Abdominal wall CCC is a rare and aggressive disease. Primary surgery of suspicious nodules arising in cesarean scars should be referred to specialized institutions. Surgical procedure should consider wide tumor resection and lymph node dissection. Adjuvant chemotherapy and radiotherapy would be recommended for potential benefits. The role of gynecological organs removal need further investigation. Whether fertility-sparing surgery could be considered is unknown. Collaborative multicenter data collection is crucial in order to provide necessary evidence for treatment modality decision.

## Supplementary Information

Supplementary Table S1.

## Data Availability

The institutional cancer database involves sensitive patient information, which are available upon request. Anyone who is interested in the information should contact docwuxh@hotmail.com or wu.xh@fudan.edu.cn.

## References

[1] Schnieber D, Wagner-Kolb D (1986). Malignant transformation of extragenital endometriosis. Geburtshilfe Frauenheilkd..

[2] Taburiaux L, Pluchino N, Petignat P, Wenger JM (2015). Endometriosis-associated abdominal wall cancer: A poor prognosis?. Int. J. Gynecol. Cancer..

[3] Ecker AM, Donnellan NM, Shepherd JP, Lee TT (2014). Abdominal wall endometriosis: 12 years of experience at a large academic institution. Am. J. Obstet. Gynecol..

[4] Giannella L, Serri M, Maccaroni E, Carpini GD, Berardi R, Sopracordevole F, Ciavattini A (2020). Endometriosis-associated clear cell carcinoma of the abdominal wall after caesarean section: A case report and review of the literature. In Vivo.

[5] Lai YL, Hsu HC, Kuo KT, Chen YL, Chen CA, Cheng WF (2019). Clear cell carcinoma of the abdominal wall as a rare complication of general obstetric and gynecologic surgeries: 15 years of experience at a large academic institution. Int. J. Environ. Res. Public Health.

[6] Mert I, Semaan A, Kim S, Ali-Fehmi R, Morris RT (2012). Clear cell carcinoma arising in the abdominal wall: Two case reports and literature review. Am. J. Obstet. Gynecol..

[7] Ruiz MP, Wallace DL, Connell MT (2015). Transformation of abdominal wall endometriosis to clear cell carcinoma. Case Rep. Obstet. Gynecol..

[8] Liu H, Leng J, Lang J, Cui Q (2014). Clear cell carcinoma arising from abdominal wall endometriosis: A unique case with bladder and lymph node metastasis. World J. Surg. Oncol..

[9] Lopes, A., Anton, C., Slomovitz, B.M., Accardo de Mattos, L., & Marino Carvalho, F. Clear cell carcinoma arising from abdominal wall endometrioma after cesarean section. *Int. J. Gynecol. Cancer* **29**(8), 1332–1335 (2019). 10.1136/ijgc-2019-000808 31451559

[10] Gentile JKA, Migliore R, Kistenmacker FJN, Oliveira MM, Garcia RB, Bin FC, Souza P, Assef JC (2018). Malignant transformation of abdominal wall endometriosis to clear cell carcinoma: Case report. Sao Paulo Med. J..

[11] Heller DS, Houck K, Lee ES, Granick MS (2014). Clear cell adenocarcinoma of the abdominal wall: A case report. J. Reprod. Med..

[12] Matsuo K, Alonsozana EL, Eno ML, Rosenshein NB, Im DD (2009). Primary peritoneal clear cell adenocarcinoma arising in previous abdominal scar for endometriosis surgery. Arch. Gynecol. Obstet..

[13] Shalin SC, Haws AL, Carter DG, Zarrin-Khameh N (2012). Clear cell adenocarcinoma arising from endometriosis in abdominal wall cesarean section scar: A case report and review of the literature. J. Cutan Pathol..

[14] Bats AS, Zafrani Y, Pautier P, Duvillard P, Morice P (2008). Malignant transformation of abdominal wall endometriosis to clear cell carcinoma: Case report and review of the literature. Fertil. Steril..

[15] Ferrandina G, Palluzzi E, Fanfani F, Gentileschi S, Valentini AL, Mattoli MV, Pennacchia I, Scambia G, Zannoni G (2016). Endometriosis-associated clear cell carcinoma arising in caesarean section scar: A case report and review of the literature. World J. Surg. Oncol..

[16] Miller DM, Schouls JJ, Ehlen TG (1998). Clear cell carcinoma arising in extragonadal endometriosis in a caesarean section scar during pregnancy. Gynecol. Oncol..

[17] Harry VN, Shanbhag S, Lyall M, Narayansingh GV, Parkin DE (2007). Isolated clear cell adenocarcinoma in scar endometriosis mimicking an incisional hernia. Obstet. Gynecol..

[18] Roy S, Hoskins P, Tinker A, Brar H, Bowering G, Bahl G (2020). Adjuvant treatment of early ovarian clear cell carcinoma: A population-based study of whole abdominal versus pelvic nodal radiotherapy. J. Natl. Compr. Cancer Netw..

[19] Ishida GM, Motoyama T, Watanabe T, Emura I (2003). Clear cell carcinoma arising in a cesarean section scar: Report of a case with fine needle aspiration cytology. Acta Cytol..

[20] Razzouk K, Roman H, Chanavaz-Lacheray I, Scotté M, Verspyck E, Marpeau L (2007). Mixed clear cell and endometrioid carcinoma arising in parietal endometriosis. Gynecol. Obstet. Invest..

