# Supplementary material for: Treatment for clear cell carcinoma of the abdominal wall at a tertiary cancer center
Source: Sci Rep. 2022 Jun 25;12:10820. doi: 10.1038/s41598-022-14917-0 (PMC9233660; doi:10.1038/s41598-022-14917-0)
Supplement: Supplementary file 1 — Supplementary Table S1. [file 41598_2022_14917_MOESM1_ESM.docx]

| **Table S1. Cases of patients with clear cell carcinoma of the abdominal wall in literature** | | | | | | | | |
| --- | --- | --- | --- | --- | --- | --- | --- | --- |
| Reference | Age | Treatment | Surgical patterns | Gyn Organs | Radiological LN | Lymph node metastasis | Recurrence pattern | Follow-up |
| Schnieber, 1986 | 40 | Surgery+RT | Tumor resection, TAH+􏺲BSO |  |  |  | NA | Death after 18 months |
| Hitti, 1990 | 46 | Surgery | Tumor resection, TAH+􏺲BSO | - |  |  | N | No relapse after 30 months |
| Miller, 1998 | 38 | Surgery+CT+RT | Tumor resection, TAH+􏺲BSO+omentectomy | - |  |  | N | No relapse after 60 months |
| Park, 1999 | 56 | Surgery+RT | Tumor resection |  |  |  | NA | - |
| Ishida, 2003 | 56 | Surgery+CT | Tumor resection |  |  |  | lung, bone, brain | Death after 24 months |
| Sergent, 2006 | 45 | Surgery | Tumor resection+BSO |  |  |  | NA | Death after 6 months |
| Alberto, 2006 | 38 | Surgery+CT+RT | Tumor resection |  |  |  | NA | - |
| Razzouk, 2007 | 46 | Surgery+CT | Tumor resection, TAH+􏺲BSO | - |  |  | Liver | Death after 6 months |
| Harry, 2007 | 55 | Surgery+RT | Tumor resection |  |  |  | N | No relapse after 18 months |
| Rust, 2008 | 42 | Surgery | Tumor resection |  |  |  | NA | - |
| Bats, 2008 | 38 | NACT+Surgery | Tumor resection, TAH+􏺲BSO+omentectomy | - |  |  | LN | Relapse after 4 months |
| Achach, 2008 | 49 | Surgery+CT | Tumor resection |  |  |  | Local recurrence | Relapse after 6 months |
| Williams, 2009 | 53 | Surgery+CT | Tumor resection, TAH+􏺲BSO,BPLND+Inguinal LND | - | + | + | Local recurrence and LN | Death after 11 months |
| Matsuo, 2009 | 37 | Surgery+CT | Tumor resection, TAH+􏺲BSO,BPLND | - | - | - | Local recurrence | Relapse after 6 months |
| Bourdel, 2010 | 43 | Surgery+CT+RT | Tumor resection,BPLND |  | + | + | LN | Death after 22 months |
| Yan, 2011 | 41 | Surgery+CT | Tumor resection |  |  |  | N | No relapse after 24 months |
| Li, 2012 | 49 | Surgery+CT | Tumor resection, TAH+􏺲BSO | - |  |  | N | No relapse after 8 months |
| Mert, 2012 | 42 | NACT+Surgery | Tumor resection, TAH+􏺲BSO,BPLND | - | - | - | N | No relapse after 1 months |
| Mert, 2012 | 51 | Surgery+RT | Tumor resection, TAH+􏺲BSO+omentectomy | - |  |  | N | No relapse after 31 months |
| Shalin, 2012 | 47 | Surgery+CT | Tumor resection, USO,BPLND | - | - | + | LN | Relapse after 5 months |
| Ijichi, 2014 | 60 | Surgery | Tumor resection |  |  |  | Local recurrence | Relapse after 8 months |
| Aust, 2015 | 47 | Surgery+CT | Tumor resection, TAH+􏺲BSO,BPLND | - | + | + | N | No relapse 10 months after CT |
| Heller, 2014 | 37 | Surgery | Tumor resection, USO,BPLND | - | - | + | local recurrence and widely metastatic disease | Relapse after 5 months |
| Liu, 2014 | 39 | Surgery+CT | Tumor resection, TAH+􏺲BSO,BPLND+Inguinal LND | - | + | + | Pelvic | Death 12 months after CT |
| Ruiz, 2015 | 41 | Surgery+CT+RT | Tumor resection, TAH+BSO+omentectomy | - |  |  | Local recurrence | Relapse 6 months after CT |
| Ruiz, 2015 | 57 | Surgery+CT+RT | Tumor resection, TAH+􏺲BSO,BPLND+Inguinal LND | - | + | + | N | No relapse after 6 cycles of chemotherapy |
| Sosa-Duràn, 2015 | 45 | Surgery | Tumor resection |  |  |  | N | No relapse after 16 months |
| Ferrandina, 2016 | 44 | NACT+Surgery | Tumor resection, TAH+􏺲BSO,BPLND+Inguinal LND | - | + | + | Liver | Death after 6 months |
| Graur, 2017 | 43 | Surgery | Tumor resection |  |  |  | N | No relapse after 36 months |
| Marques, 2017 | 47 | Surgery+CT | Tumor resection,BSO | - |  |  | Local recurrence | No death after 45 months |
| Gentile, 2018 | 42 | Surgery+CT | Tumor resection, BSO,BPLND+Inguinal LND | - | - | + | N | No relapse after 8 months |
| Rivera Rolon, 2019 | 48 | Surgery+CT | Tumor resection |  |  |  | N | - |
| Lopes, 2019 | 48 | Surgery+CT | Tumor resection, TAH+􏺲BSO,BPLND+Inguinal LND | - | + | + | N | No relapse after 4 cycles of chemotherapy |
| Behbehani, 2019 | 48 | Surgery | Tumor resection |  |  |  | NA | - |
| Lai, 2019 | 52 | Surgery | Tumor resection, TAH+􏺲BSO | - |  |  | LN, bone | DFS=10 months, OS=13 months |
| Lai, 2019 | 56 | Surgery+CT | Tumor resection, TAH+􏺲BSO,BPLND | - | + | + | LN | DFS=3 months, OS=11months |
| Lai, 2019 | 52 | Surgery+CT | Tumor resection, TAH+􏺲BSO | - |  |  | N | DFS=93 months, OS=97 months |
| Lai, 2019 | 56 | Surgery+CT | Tumor resection, TAH+􏺲BSO,BPLND | - | - | - | NA | OS=5 months |
| Lai, 2019 | 55 | NACT+Surgery | Tumor resection, TAH+􏺲BSO+omentectomy | - |  |  | PD | OS=23 months |
| Lai, 2019 | 45 | CT+RT |  |  |  |  | PD | OS=7 months |
| Giannella, 2020 | 45 | CT+RT |  |  |  |  | PD | Death after 7 months |
| CT, chemotherapy; RT, radiotherapy; NACT, neoadjuvant chemotherapy; TAH, total abdominal hysterectomy; BSO, bilateral salpingo-oophorectomy; Abd, abdominal; Gyn, Gynecological organs; LN, lymph node; BPLND, bilateral pelvic lymph node dissection; NA, not available; PD, progressive disease; DFS, disease-free survival; OS, overall survival | | | | | | | | |
|  |  |  |  |  |  |  |  |  |
